# Determinants of multidrug‐resistant tuberculosis in São Paulo—Brazil: a multilevel Bayesian analysis of factors associated with individual, community and access to health services

**DOI:** 10.1111/tmi.13409

**Published:** 2020-05-28

**Authors:** Luiz Henrique Arroyo, Mellina Yamamura, Antônio Carlos Vieira Ramos, Laura Terenciani Campoy, Juliane de Almeida Crispim, Thais Zamboni Berra, Luana Seles Alves, Yan Mathias Alves, Felipe Lima dos Santos, Ludmilla Leidianne Limirio Souza, Alexandre Tadashi Inomata Bruce, Hamilton Leandro Pinto de Andrade, Valdes Roberto Bollela, Elias Teixeira Krainski, Carla Nunes, Ricardo Alexandre Arcêncio

**Affiliations:** ^1^ Ribeirão Preto College of Nursing University of São Paulo Ribeirão Preto Brazil; ^2^ Department of Nursing Federal University of São Carlos São Carlos Brazil; ^3^ Ribeirão Preto Medical School University of São Paulo São Paulo Brazil; ^4^ Department of Statistics Federal University of Paraná Curitiba Brazil; ^5^ National School of Public Health Nova University of Lisbon Lisbon Portugal

**Keywords:** multidrug‐resistant tuberculosis, risk factors, communicable disease control, socioeconomic factors, health care quality, access, and evaluation, tuberculose multirésistante, facteurs de risque, lutte contre les maladies transmissibles, facteurs socioéconomiques, qualité, accès et évaluation des soins de santé

## Abstract

**Objective:**

Multidrug‐resistant tuberculosis (MDR‐TB) remains a serious public health problem worldwide. Accordingly, this study sought to identify individual, community and access to health services risk factors for MDR‐TB.

**Methods:**

Retrospective cohort of all TB cases diagnosed between 2006 and 2016 in the state of São Paulo. A Bayesian spatial hierarchical analysis with a multilevel design was carried out.

**Results:**

It was identified that the history of previous TB treatment (Odds Ratios [OR]:13.86, 95% credibility interval [95% CI]:12.06–15.93), positive sputum culture test (OR: 5.26, 95% CI: 4.44–6.23), diabetes mellitus (OR: 2.34, 95% CI: 1.87–2.91), residing at a standard address (OR: 2.62, 95% CI: 1.91–3.60), positive sputum smear microscopy (OR: 1.74, 95% CI: 1.44–2.12), cavitary pulmonary TB (OR: 1.35, 95% CI: 1.14–1.60) and diagnosis performed due to spontaneous request (OR: 1.26; 95% CI: 1.10–1.46) were associated with MDR‐TB. Furthermore, municipalities that performed HIV tests in less than 42.65% of patients with TB (OR: 1.50, 95% CI: 1.25–1.79), that diagnosed TB cases only after death (OR: 1.50, 95% CI: 1.17–1.93) and that had more than 20.16% of their population with income between ¼ and ½ of one minimum wage (OR: 1.56, 95% CI: 1.30–1.87) were also related to the MDR‐TB.

**Conclusions:**

Knowledge of these predictive factors may help to develop more comprehensive disease prevention strategies for MDR‐TB, avoiding the risks expressed regarding drug resistance expansion.

## Abstract (fr)

**Objectif:**

La tuberculose multirésistante (TB‐MDR) reste un grave problème de santé publique dans le monde. Cette étude visait à identifier les facteurs de risque individuels, communautaires et d'accès aux services de santé pour la TB‐MDR.

**Méthodes:**

Analyse de cohorte rétrospective de tous les cas de TB diagnostiqués entre 2006 et 2016 dans l'Etat de São Paulo par analyse bayésienne spatiale à plusieurs niveaux.

**Résultats:**

Les antécédents de traitements antituberculeux (Rapports de cotes [OR]: 13,86, Intervalle de confiance à 95% [IC95%]: 12.06‐15.93), un test de culture d'expectorations positif (OR: 5,26, IC95%: 4,44‐6,23), le diabète sucré (OR: 2,34, IC95%: 1,87‐2,91), la résidence à une adresse standard (OR: 2,62, IC95%: 1,91‐3,60), la microscopie à frottis positif (OR: 1,74, IC95%: 1,44‐2,12), la TB pulmonaire (OR: 1,35, IC95%: 1,14‐1,60) et le diagnostic réalisé en raison d'une demande spontanée (OR: 1,26; IC95%: 1,10‐1,46) étaient associés à la TB‐MDR. Les municipalités qui ont effectué des tests de dépistage du VIH chez moins de 42,65% des patients atteints de TB (OR: 1,50, IC95%: 1,25‐1,79), qui ont diagnostiqué des cas de TB uniquement après le décès (OR: 1,50, IC95%: 1,17‐1,93) et qui avaient plus de 20,16% de leur population avec un revenu entre ¼ et ½ d'un salaire minimum (OR: 1,56, IC95%: 1,30‐1,87) étaient également associées à la TB‐MDR.

**Conclusions:**

La connaissance de ces facteurs prédictifs peut aider à développer des stratégies plus complètes de prévention des maladies pour la TB‐MDR, en évitant les risques d'extension de la résistance aux médicaments.

## Introduction

Tuberculosis (TB) is a serious public health problem for millions of people every year and is one of the leading causes of death worldwide. Estimates from the World Health Organization (WHO) indicate that 10 million new cases of the disease occurred in 2018, of which approximately 400 000 people had multidrug‐resistant tuberculosis (MDR‐TB), which is defined as resistance in at least two of the main medications used in the treatment of TB, isoniazid and rifampicin [1].

MDR‐TB cases require bacteriological confirmation through drug sensitivity testing, which is not always universally available to the population. In addition, its treatment is more complex when compared to those with drug‐sensitive TB (TBs) due to the need for the administration of second‐line drugs for periods ranging from 9 to 20 months and the increased occurrence of serious adverse events [2].

As a result, treatment success is relatively low, the average care costs of MDR‐TB sufferers are up to six times higher and only one in three are diagnosed and start treatment [1]. Given the complexity of drug resistance and the risks of it spreading, it can be considered a threat to the achievement of both the End‐TB Strategy and the Millennium Sustainable Development goals and targets, calling for the intensification of global efforts to address its prevention [2].

In Brazil, in 2016, there were 1044 drug‐resistant cases, among which 25.7% were diagnosed with MDR‐TB [3]. In the subnational context, the state of São Paulo, one of Brazil's main economic and political regions, is considered a key scenario for TB prevention and care, as it has the largest absolute number of new TB cases and second largest number of MDR‐TB cases in the country [4].

It is important to mention that there are few studies conducted in the Brazilian scenario on MDR‐TB, resulting in a significant gap in knowledge about the disease. In addition, identifying risk factors for MDR‐TB in different regions of the world and with different epidemiological scenarios may help to develop more comprehensive disease prevention strategies for each context [5]. Therefore, this study aimed to identify the determinants of MDR‐TB considering individual, community factors and access to health services in São Paulo state, Brazil.

## Methods

### Study design and scenario

This is a retrospective cohort study conducted in 645 municipalities in São Paulo state, one of the 27 federal units in Brazil. The São Paulo state is located in the southeastern region of the country, with an estimated population of 45 million inhabitants, representing approximately 22% of the entire Brazilian population [6].

### Reference population, data collection and analysis

**Figure 1:**
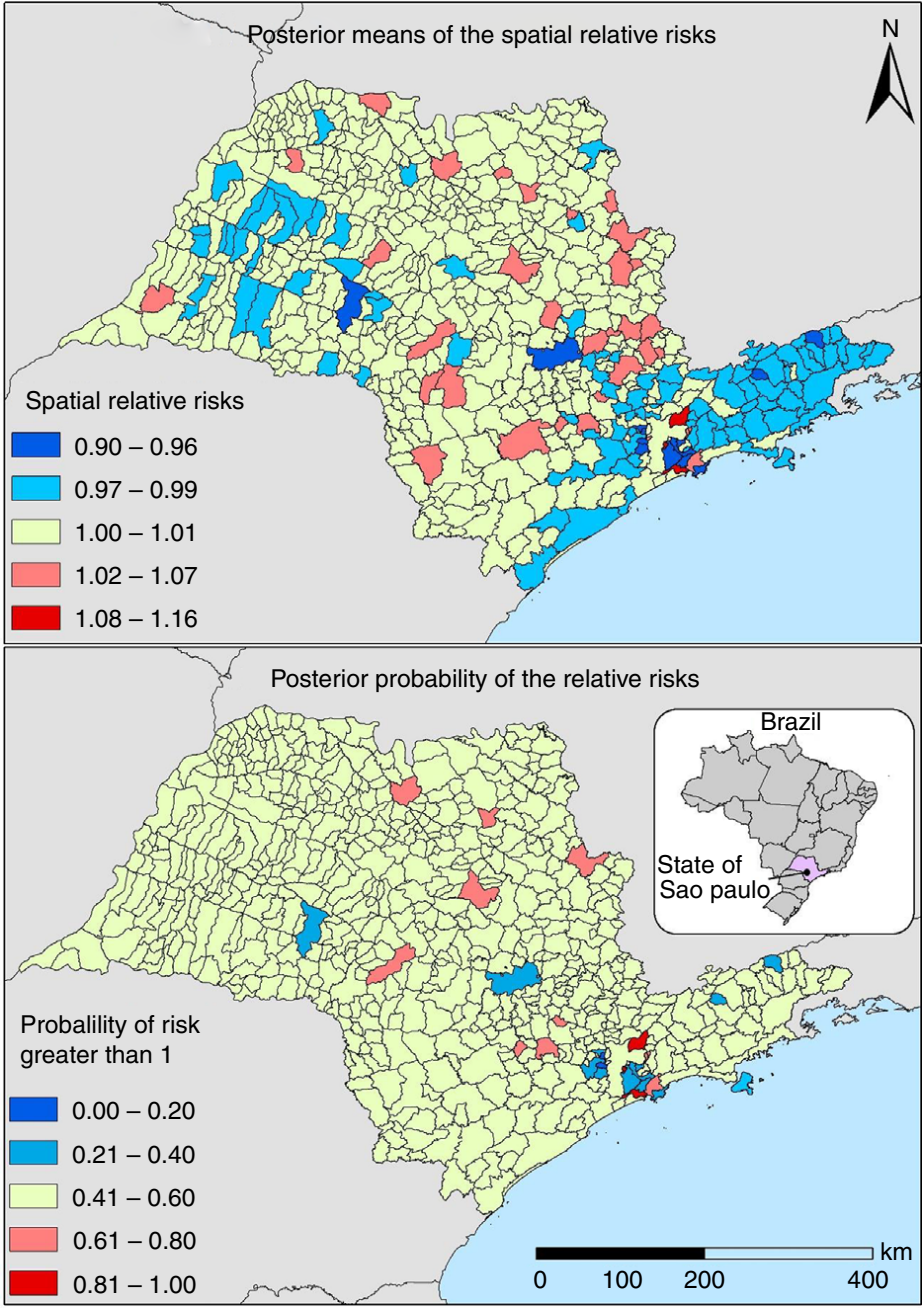
Map of the mean posteriori distribution of relative risk and later likelihood that relative risks are >1 for multidrug‐resistant tuberculosis, São Paulo, Brazil, 2006 to 2016.

The population consisted of confirmed TB cases, whether TBs or MDR‐TB, registered in the Tuberculosis Patient Control System (TB‐WEB) between 2006 and 2016, residing in São Paulo municipalities at the time of diagnosis (Figure 1).

Regarding the classification of the municipality of residence, those who resided there at the time of their diagnosis were considered, regardless of whether the treatment had been given in other locations or if a change of residence had occurred. In the case of those deprived of liberty, the municipality of residence was classified using the location of the prison unit.

The following exclusion criteria were adopted: the absence of filling in the place of residence in the registration form; and registrations presenting other forms of TB resistance. Cases considered to be duplicated were also excluded. The individual's full name, the full name of the mother and date of birth of the individuals were used as reference.

For TB cases, the most recent registrations in TB‐WEB were included in the analytical steps, while for MDR‐TB cases, the first registration with this diagnosis was used, regardless of whether they had subsequent records. The justification for this choice was given by the very objective proposed for the study, since registrations from future periods could be a consequence of other conditions.

In order to analyse the determining factors for the occurrence of MDR‐TB, variables that expressed the individual, community and access to health services were considered. These factors are in line with the context of social determinants of health, according to the conceptual framework elaborated by WHO [7].

Considering this theoretical framework, a multilevel analysis structure was designed relating individual population data and aggregated variables (ecological level) that characterised the municipalities of residence of the individuals. This information was collected through different information systems and official publications of the Brazilian government, as presented in Table 1.

**Table 1:** Variables listed for analysis, description, method of obtaining and data source

Analytical Dimension	Variable	Indicator description	Period	Data source
Dependent variable (response)				
Individual level	Sensitive Tuberculosis/Multidrug‐resistant tuberculosis	Diagnosis on registration form	2006–2016	TB‐WEB
Independent Variables (explanatory)				
Individual level	Previous History of Tuberculosis Treatments	Yes/No	2006–2016	TB‐WEB
	Ethnicity	White/Black/Brown/Other/No Information	2006–2016	TB‐WEB
	Age	≤14 years/15–30 years/31 to 59 years/≥60 years	2006–2016	TB‐WEB
	Sex	Male/Female	2006–2016	TB‐WEB
	Schooling	≤7 years of study/>7 years of study/No information	2006–2016	TB‐WEB
	Date of diagnosis	Year (2006–2016)	2006–2016	TB‐WEB
	Clinical type of tuberculosis	Pulmonary/Extrapulmonary	2006–2016	TB‐WEB
	Diagnostic Form	Through patient's own demand/Active case search by health service	2006–2016	TB‐WEB
	Sputum smear examination result	Positive/Negative/No Information	2006–2016	TB‐WEB
	Culture examination result	Positive/Negative/Not Performed/No Information	2006–2016	TB‐WEB
	X‐ray examination result	Non‐Cavity/Cavity/No Information	2006–2016	TB‐WEB
	Acquired Immunodeficiency Syndrome (AIDS)	Yes/No	2006–2016	TB‐WEB
	Diabetes mellitus	Yes/No	2006–2016	TB‐WEB
	Alcoholism	Yes/No	2006–2016	TB‐WEB
	Mental disease	Yes/No	2006–2016	TB‐WEB
	Illicit drug use	Yes/No	2006–2016	TB‐WEB
	Smoking	Yes/No	2006–2016	TB‐WEB
	Place of residence	Standard residence/Deprived of liberty/No fixed residence	2006–2016	TB‐WEB
Aggregate Level (data of the municipality of residence)	Total inhabitants	Below/Above State Median (median = 13 059.5 inhabitants)	2010	2010 Census (IBGE)
	Degree of urbanisation	Below/Above State Median (median = 90.43%)	2010	2010 Census (IBGE)
	Demographic density	Below/Above State Median (median = 38.82 inhab./km^2^)	2010	2010 Census (IBGE)
	Municipality with prison unit	Yes/No	2018	Secretariat of Penitentiary Administration of SP
	Human development Index	Below/Above State Median (median = 0.73)	2010	2010 Census (IBGE)
	Proportion of people with access to water supply	Below/Above State Median (median = 99.01%)	2010	2010 Census (IBGE)
	Proportion of people with access to garbage collection service	Below/Above State Median (median = 99.72%)	2010	2010 Census (IBGE)
	Proportion of people with access to sewage service	Below/Above State Median (median = 96.89%)	2010	2010 Census (IBGE)
	Average Gross Domestic Product in the period	Below/Above the state median (median = $ 19 699.05)	2006–2016	IBGE
	Proportion of population with per capita monthly nominal income of ¼ to ½ minimum wage	Below/Above the state median (median = 20.16%)	2010	2010 Census (IBGE)
	Proportion of population with per capita monthly nominal income up to ¼ minimum wage	Below/Above State Median (median = 5.28%)	2010	2010 Census (IBGE)
	Gini Index	Below/Above State Median (median = 0.40)	2010	2010 Census (IBGE)
	Average Coverage of *Bolsa Famíli*a Program in the period	Below/Above State Median (median = 8.99%)	2006–2016	Senarc (Ministry of Citizenship) ‐ Single Registry
	Average coverage of Primary Care in the period	Below/Above State Median (median = 89.97%)	2006–2016	National Register of Health Facilities (CNES)
	Average coverage of the Family Health Strategy in the period	Below/Above State Median (median = 54.18%)	2006–2016	National Register of Health Facilities (CNES)
	Proportion of treatment dropouts in the period	Below/Above State Median (median = 5.91%)	2006–2016	TB‐WEB
	Cases diagnosed after death in the period	No/Yes	2006–2016	TB‐WEB
	Proportion of Sensitivity Tests performed in the period	Below/Above State Median (median = 0%)	2006–2016	TB‐WEB
	Proportion of Directly Observed Treatments performed during the period	Below/Above State Median (median = 60.46%)	2006–2016	TB‐WEB
	Proportion of cases diagnosed in Accident and Emergency and/or Hospitalisation in the period	Below/Above State Median (median = 10%)	2006–2016	TB‐WEB
	Proportion of cases diagnosed by active search by health services in the period	Below/Above State Median (median = 1.04%)	2006–2016	TB‐WEB
	Proportion of sputum smear microscopy performed in the period	Below/Above State Median (median = 41.40%)	2006–2016	TB‐WEB
	Proportion of cultures performed in the period	Below/Above State Median (median = 14.28%)	2006–2016	TB‐WEB
	Proportion of human immunodeficiency virus (HIV) tests performed during the period	Below/Above State Median (median = 42.65%)	2006–2016	TB‐WEB
	Average public expenditure invested in health per inhabitant in the period	Below/Above State Median (median = R $ 610.67 per inhabitant)	2007–2013	SEADE Foundation
	Average number of nurses per thousand inhabitants in the period	Below/Above State Median (median = 2.86 per 1000 population)	2013–2016	SEADE Foundation
	Average number of doctors per thousand inhabitants in the period	Below/Above State Median (median = 1.99 per 1000 population)	2013–2016	SEADE Foundation
	Average number of nursing technicians per thousand inhabitants in the period	Below/Above State Median (median = 1.18 per 1000 population)	2013–2016	SEADE Foundation
	Average number of nursing assistants per thousand inhabitants in the period	Below/Above State Median (median = 3.10 per 1000 population)	2013–2016	SEADE Foundation

### Statistical analysis

Initially, descriptive analyses of the TBs and MDR‐TB cases were performed by describing their sociodemographic and clinical–operational characteristics. In order to identify the determinants associated with MDR‐TB, data were dichotomised between TBs and MDR‐TB cases. Assuming that the response variable followed a binomial distribution, a logistic regression was performed considering the spatial hierarchical Bayesian approach. The fixed effects of this analysis are presented in Table 1, which are both at the individual and household level (municipality of residence).

We observed that, for some individual variables gathered from TB‐WEB information system, there was not its complete filling for some patients. These variables were ethnicity, education, sputum test, culture test and radiography. To handle this situation during the analysis, we added the missing data as one of the categories of the explanatory variables.

The year of diagnosis of the individual was incorporated as a discrete variable (2006 to 2016), that is, one of the fixed effects of the model. At the aggregate level, the variables of the municipalities of residence of those affected by the disease were dichotomised considered as the cut‐off criterion for the state median.

Due to the large number of variables proposed in the explanatory model, a selection of these variables was performed by the stepwise method (backward elimination) through the Bayesian information criterion (BIC), which used statistical significance values (*P* value) <0.1 for the inclusion of variables in the multiple logistic regression model as a criterion. Next, the final model was selected from the lowest Watanabe–Akaike information criterion (WAIC) value identified by the backward elimination method. It is important to note that the present method of selecting variables has its own limitations. Since that are no agreement on the best criterion for the addition and deletion of variables in a stepwise procedure, it is possible that the non‐significant variables, when taken in aggregate, may have important information, mainly if there are correlated covariates. Despite this, our goal was to find the best fitting and most parsimonious, yet biologically reasonable model to describe the relationship between the response variable and the set of independent variables (Heinze; Wallisch; Dunkler, 2017).

After the construction of the statistical model with fixed effects, the area‐specific effect was incorporated as a random effect. This distribution was introduced considering the Besag, York and Mollié model [8]. This model allows the calculation of relative risk (RR) from the posterior distribution of spatial analysis units.

The Jenks Natural Breaks method was considered as a criterion for interval construction [9] for maps with RR representations. An non‐informative priori was considered in the analyses, and a posteriori distribution was obtained using the Integrated Nested Laplace Approximation (INLA) in the R‐INLA package [10]. Odds ratios (OR) and credible intervals were obtained in 95% (95% CI) of posteriori distributions. The analyses were performed using the R software, and the maps were prepared using ArcGis 10.6.

### Ethical aspects

The study was approved by the Human Ethics Committee of the University of São Paulo at Ribeirão Preto College of Nursing (CAAE 99805318.0.0000.5393).

## Results

There were 194 251 cases of diagnosed TB and, after removing the duplicates, 167 726 cases remained, among which 866 were diagnosed as MDR‐TB. The characteristics of those diagnosed with the sensitive and resistant form of the disease are presented in Table 1.

The predominant epidemiological profile between TBs and MDR‐TB was relatively similar, with a higher prevalence of cases among white individuals, between 31 and 59 years old, male, with seven years of schooling and pulmonary disease. Regarding the clinical examinations, the cases diagnosed with MDR‐TB performed TB tests more frequently, this is a constant for all three examinations analysed in the present investigation.

It was observed that the diagnosis through active search was less usual and the most frequent comorbidities in both groups were alcoholism and acquired immunodeficiency syndrome (AIDS). In relation to the place of residence, living in a standard dwelling was the most recurring feature.

In order to identify the determinants of MDR‐TB in relation to sensitive TB cases, the explanatory variables were initially selected to be included into the model by the stepwise method and BIC criterion. Among the 52 initial variables determined for the analysis, 21 were selected for insertion into the multiple model (Table S1). The final model shows its results with and without the spatial effect, as presented in Table 2.

**Table 2:** Characteristics of cases of sensitive and multidrug‐resistant tuberculosis in the state of São Paulo, 2006 to 2016, Brazil

Variable	Sensitive tuberculosis *n* (%)	Multidrug‐resistant tuberculosis *n* (%)
Previous history of tuberculosis		
No	155 363 (93%)	392 (45.3%)
Yes	11 497 (6.8%)	474 (54.7%)
Ethnicity		
White	63 999 (38.5%)	375 (43.3%)
Black and brown	55 131 (33%)	289 (33.4%)
Others	2172 (1.3%)	6 (0.7%)
Uninformed	45 558 (27.3%)	196 (22.6%)
Age		
Up to 14 years	5360 (3.2%)	8 (1%)
15–30 years	56 776 (34%)	274 (31.6%)
31–59 years old	85 587 (51.3%)	527 (60.8%)
60 years or older	19 137 (11.5%)	57 (6.6%)
Sex		
Male	114 627 (68.7%)	612 (70.7%)
Feminine	52 233 (31.3%)	254 (29.3%)
Schooling		
≤7 years of study	67 500 (40. 4%)	388 (44.8%)
>7 years of study	58 533 (35.1%)	328 (37.9%)
Uninformed	40 827 (24.5%)	150 (13.3%)
Clinical Form		
Pulmonary	139 186 (83.4%)	837 (96.6%)
Extrapulmonary	27 674 (16.6%)	29 (3.4%)
Discovery form		
No active search	96 957 (58.1%)	540 (60.4%)
Active Search	69 902 (41.9%)	326 (39.6%)
Sputum smear examination result		
Positive	90 716 (54.4%)	676 (78.1%)
Negative	43 991 (26.3%)	136 (15.7%)
Uninformed	32 153 (19.3%)	54 (6.2%)
Culture examination result		
Positive	41 818 (25.1%)	538 (62.2%)
Negative	21 472 (12.9%)	54 (6.2%)
Not performed	83 322 (50%)	215 (24.8%)
Uninformed	20 248 (12.1%)	59 (6.8%)
X‐ray examination result		
Non‐cavity	105 346 (63.1%)	532 (61.4%)
Cavity	25 174 (15.1%)	213 (24.6%)
Uninformed	36 340 (21.8%)	121 (14%)
AIDS		
Positive	16 265 (9.7%)	106 (12.2%)
Negative	150 595 (90.3%)	760 (87.8%)
Diabetes mellitus		
Yes	9195 (5.5%)	99 (11.4%)
Not	157 665 (94.5%)	767 (88.6%)
Alcoholism		
Yes	22 899 (13.7%)	175 (20.2%)
Not	143 961 (86.3%)	691 (79.8%)
Mental disease		
Yes	2778 (1.7%)	9 (1%)
Not	164 082 (98.3%)	857 (99%)
Illicit drug use		
Yes	13 423 (8%)	80 (9.2%)
Not	153 437 (92%)	786 (90.8%)
Smoking		
Yes	11 605 (7%)	55 (7%)
Not	155 255 (93%)	811 (93%)
Place of residence		
Standard Residence	147 185 (88.2%)	799 (92.3%)
Deprived of liberty	15 998 (9.6%)	50 (5.7%)
No fixed residence	3677 (2.2%)	17 (2%)

The final result with the spatial component was relatively better than that without the spatial effect, given the reduction of the WAIC value. Regarding the variables at the individual level, presenting previous history of TB (OR: 13.86; 95% CI: 12.06–15.93), positive culture examination (OR: 5.26; 95% CI: 4.44–6.23) and smear microscopy (OR: 1.74; 95% CI: 1.44–2.12), diagnosed with diabetes mellitus (OR: 2.34; 95% CI: 1.87–2.91), reside at standard housing (OR: 2.62; 95% CI: 1.91–3.60), X‐ray examination with detection of pulmonary cavities (OR: 1.35; 95% CI: 1.15–1.60) and being diagnosed without active search (OR: 1.26; 95% CI: 1.09–1.46) were factors that increased the chances of developing MDR‐TB (Table 3).

**Table 3:** A posteriori distribution of fixed effects with and without spatial component of logistic regression analysis of determinants of multidrug‐resistance tuberculosis in the state of São Paulo, Brazil, 2006 to 2016

Variable		
Fixed effect	Odds ratio (95% CI) Model without component Space*	Odds ratio (95% CI) Component model Spatial†
Previous history of tuberculosis		
No	1	1
Yes	13.91 (12.10–15.99)	13.86 (12.06–15.93)
Culture examination result		
Not performed	1	1
Positive	5.24 (4.42–6.20)	5.26 (4.44–6.23)
Negative	1.24 (0.91–1.69)	1.24 (0.91–1.69)
Uninformed	0.88 (0.65–1.18)	0.86 (0.64–1.16)
Year of diagnosis (discrete type)	0.89 (0.87–0.91)	0.89 (0.87–0.91)
Diabetes mellitus		
Negative	1	1
Positive	2.34 (1.87–2.91)	2.34 (1.87–2.91)
Place of residence		
Deprived of liberty	1	1
No fixed residence	0.91 (0.52–1.62)	0.92 (0.52–1.63)
Standard Residence	2.60 (1.89–3.56)	2.62 (1.91–3.60)
Proportion of HIV tests performed (ecological variable)		
≤42.65%	1	1
>42.65%	1.47 (1.26–1.72)	1.50 (1.25–1.80)
Sputum smear examination result		
Negative	1	1
Positive	1.75 (1.44–2.12)	1.74 (1.44–2.12)
Uninformed	0.97 (0.69–1.34)	0.98 (0.70–1.34)
X‐ray examination result		
Non‐cavity	1	1
Cavity	1.35 (1.14–1.59)	1.35 (1.15–1.60)
Uninformed	0.68 (0.55–0.84)	0.68 (0.55–0.85)
Cases diagnosed after death (ecological variable)		
No	1	1
Yes	1.51 (1.19–1.93)	1.50 (1.17–1.93)
Proportion of population with per capita monthly nominal income of ¼ to ½ minimum wage (ecological variable)		
≤20.16%	1	1
>20.16%	1.57 (1.34–1.84)	1.57 (1.30–1.88)
Discovery Form		
Active Search	1	1
No active search	1.26 (1.09–1.46)	1.26 (1.09–1.46)
Random Effect (Spatial Component)		Mean posterior distribution (95% CI)
Precision for analysis unit	–	73.02 (72.99–73.06)
Φ for analysis unit	–	0.11 (0.10–0.12)

* Watanabe–Akaike information criterion (WAIC): 8636.69.

† Watanabe–Akaike information criterion (WAIC): 8624.78.

Two individual variables had a protective effect on the occurrence of MDR‐TB. The first was the lack of completion in the X‐ray examination notification form (OR: 0.68; 95% CI: 0.55–0.85), and the second was the individual's year of diagnosis. Over the period analysed, the chance of developing MDR‐TB relative to sensitive TB decreased by 11% (OR: 0.89; 95% CI: 0.87–0.91).

Regarding community factors and access to health services at the ecological level, it was observed that the proportion of human immunodeficiency virus (HIV) tests performed among patients with TB less than or equal to 42.65% (OR: 1.50; 95% CI: 1.25–1.80), have cases diagnosed after death (OR: 1.50; 95% CI: 1.17–1.93) and have a ratio higher than 20.16% in the municipal population with a monthly per capita nominal income of ¼ to ½ minimum wage (OR: 1.57; 95% CI: 1.30–1.88), increasing the chances of MDR‐TB in those residents in these locations.

From the mean posteriori distribution of the spatial random effect, spatial RR maps of the municipalities were developed for the occurrence of MDR‐TB. The spatial RR ranged from 0.90 to 1.16, and among the 645 municipalities in the state, 28 (4.3%) had a risk of MDR‐TB (RR = 1.02 to 1.16), 285 (44.2%) had protective characteristics for the disease (RR = 0.90 to 0.99), and the predominance of the municipalities (*n* = 332, 51.5%) was not associated with the occurrence of MDR‐TB (RR = 1.00–1.01).

To understand the uncertainties of the event in question, the RR probability map was greater than one was prepared, that is, the chance of the municipality presenting a risk for MDR‐TB. It was found that the areas considered at risk did not tend to form clusters in space.

## Discussion

The study aimed to identify the determinants of MDR‐TB in the São Paulo state, a pioneering research considering its multilevel Bayesian spatial methodology, which allowed the incorporation of individual, community factors and access to health services.

It was identified that both individual and community factors and access to health service were associated with the occurrence of MDR‐TB. The results confirmed previous evidence regarding the history of previous TB treatment, which is most likely the most prominent risk condition for MDR‐TB development, judging by its effect regardless of geographic context [5].

In the study, individuals who had a previous history of treatment represented those who completely underwent treatment, failed or did not adhere, meaning that in order to prevent the development of MDR‐TB, it is necessary to consider variations in adherence to TB treatment, in the pharmacokinetic profile of the drugs used and the pharmacogenetics of those affected by TB [5, 11, 12].

The results highlight the need to improve the care provided to individuals diagnosed with TB by ensuring the optimal drug dose, effective and authentic drug use, individual‐centred care, improved quality of care and access to drug sensitivity testing [13]. In addition, it is important to consider the monitoring actions of therapeutic interventions with an experienced multiprofessional team in order to achieve greater chances of treatment success [14].

*Diabetes mellitus* was the only one of the comorbidities analysed to be associated with the development of MDR‐TB, a finding that corroborates previous studies [15, 16]. There is ample evidence that diabetes mellitus is a risk factor for TB infection [17], but the reasons for this increase in the risk of MDR‐TB are still poorly understood and findings in the scientific literature are quite controversial. Some hypotheses highlight that a possible slower response to TB drug treatment and compromised immune system, which facilitates the occurrence of infections in the body, along with other prevalent health problems, such as peripheral nerve damage and reduced blood flow to extremities could be risk factors [18].

The authors highlight that individuals with both diseases should be closely monitored for dosage and drug regimen adjustments; and that anti‐TB treatment should be reviewed and differentiated from those diagnosed with TB alone in order to avoid drug resistance [19]. Given a global scenario of the diabetes mellitus epidemic [20] and the high incidence of TB in many countries [1], recognising this risk may be essential for MDR‐TB prevention actions. However, it is important to emphasise the need for further investigations to understand this relationship more clearly and how control interventions can be implemented.

In this study, the place of residence was evaluated considering whether the individual was in prison, homeless or living in ordinary accommodation. As a result, it was identified that living in ordinary accommodation was related to the occurrence of MDR‐TB cases, which contradicts some studies [21, 22]. However, according to Pradipta et al., the relationship of housing with drug resistance may vary greatly depending on the context in which it is analysed. As an example, in a study conducted in London, high TB resistance rates were commonly related to the living conditions of homeless and people deprived of liberty, and however, this population subgroup accounted for approximately half of the country's total patients [23], a highly differentiated profile from the one the present study proposed to analyse, which presented less than 8% of MDR‐TB cases.

Consequently, the present results may reflect a specific MDR‐TB scenario in which the health care provided to this population subgroup is of a different quality, to the point of avoiding the occurrence of MDR‐TB; or that those who live on the streets or in prisons are not diagnosed in a timely manner, resulting in the underreporting of MDR‐TB cases [24].

Another point observed as a risk factor for MDR‐TB was the identification of cases, and that diagnosis other than from active search represented a risk for the outcome in question. This means that individuals detected in emergency units, through hospitalisations or even spontaneous demand were more likely to develop resistance to anti‐TB drugs. Thus, there is a hypothesis that there is a difficulty in the timely detection of TB cases in the state, either due to difficulties in identifying the symptoms of the disease, lack of diagnostic resources, deficiencies in access to health actions and services or low sensitivity of the health surveillance system [25].

The final explanatory model showed the risk of MDR‐TB occurrence in relation to the results of TB diagnostic tests. The positive culture test had the highest risk for outcome among the diagnostic forms analysed. This test is characterised by both the high specificity and sensitivity for bacteriological confirmation of TB compared to direct sputum microscopy as well as a reference (gold standard) in phenotypic testing for drug susceptibility testing [2]. However, performing the culture requires adequate logistic organisation for the collection, storage and transport of samples, as well as specific laboratory resources to prevent contamination and enable proper bacterial growth, which can often require up to eight weeks [26].

The access to this technology is not always equally available to the population, which may be one of the reasons for identifying a predominance of the test for individuals with MDR‐TB, given the 30% difference compared to TBs. This indicates that health professionals recommend culture testing to individuals with more critical clinical conditions or a history of previous TB treatment. In Brazil, it is recommended that culture for mycobacteria with antimicrobial susceptibility testing is performed in all diagnosed or suspected of TB cases, something which the present study may present as a limitation in the state [26].

Positive sputum smear microscopy and X‐ray with the presence of pulmonary cavities were also related to MDR‐TB. Bacilloscopy was predominantly used for TB diagnosis, probably because it is a simple, safe method that can detect 60% to 80% of pulmonary TB cases [26].

On the other hand, chest X‐ray imaging is an appropriate method for initial assessment and follow‐up of TB cases, as it enables the identification of radiological patterns suggestive of the disease, such as the pulmonary cavities caused by extensive caseous necrosis as a result of the pathogen's own action [27]. The relationship between the pathological process of lung cavitation and the development of MDR‐TB has been reported in previous research [28, 29], including the higher prevalence of this event in people who have TB and diabetes mellitus [30].

The association of the three diagnostic tests with the analysed outcome may be related to the higher degree of infectivity and bacillary burden to which individuals with MDR‐TB present, resulting in greater positivity in the bacteriological examinations and high chances of the processes causing cavities in the lungs [27, 31].

The characteristic of the municipalities of residence of the studied population was also related to MDR‐TB. Regarding the actions for the prevention and control of TB, municipalities with HIV testing below the state average and which diagnosed cases via necropsy were more likely to present cases of resistance.

Routine HIV testing for individuals diagnosed with TB is admittedly important for disease control, regardless of the epidemiological context. Barriers to testing in municipalities may vary, but integration between HIV and TB testing and counselling services can improve the coverage of testing [32].

In a study that performed autopsies of patients admitted to a tertiary service, it was possible to show that the MDR‐TB cases were mostly undiagnosed, reflecting the difficulty in detecting these cases [33]. Both variables, which consider access to health services, indicate some fragility in the TB care network in the municipalities, which may represent a risk for the emergence of MDR‐TB.

In terms of contextual and or community variables, there was an increased risk of MDR‐TB when considering a proportion of the municipality's inhabitants with income between ¼ and ½ of the minimum wages (equivalent to US$240 to US$485 per month), meaning that the higher the number of people with low income, the higher the chances of resistance. This finding corroborates previous evidence [34, 35], reaffirming the relevance of social determinants of health for MDR‐TB and the importance of social protection as an intervention [36].

The spatial distribution of MDR‐TB showed a mild capacity to explain the occurrence of the disease in the state. This may be an effect of the small transmission of resistant bacillus between municipalities, representing an autochthonous transmission chain of the disease. This finding agrees with results found in Portugal [37] and Brazil [38].

The study has limitations regarding its research design, in which secondary data collected in a health information system may have inaccuracies with the epidemiological reality of TB. Also related to this, considering the complexity involved in the diagnosis of MDR‐TB, underreporting may occur frequently.

In conclusion, preventing, controlling, monitoring, investigating and monitoring MDR‐TB growth is a global priority and requires interventions to address its determinants, which the analyses conducted in this study were able to highlight across a broad spectrum of individual, community variables, and access to health services. Thus, TB elimination goals can be achieved in a timely manner, avoiding the risks expressed regarding drug resistance expansion.

## Funding

This work was supported by: Fundação de Amparo à Pesquisa do Estado de São Paulo – São Paulo Research Foundation (FAPESP) grant number 2017/11040‐4 and grant number 2018/14337‐0.

## Supporting information


**Table S1.** Bayesian Information Criterion results and significance values for variable selection for multivariate logistic model using the stepwise method.
