# Supplementary material for: Determinants of multidrug‐resistant tuberculosis in São Paulo—Brazil: a multilevel Bayesian analysis of factors associated with individual, community and access to health services
Source: Trop Med Int Health. 2020 May 28;25(7):839–49. doi: 10.1111/tmi.13409 (PMC7383622; doi:10.1111/tmi.13409)
Supplement: Supplementary file 1 — Table S1. Bayesian Information Criterion results and significance values for variable selection for multivariate logistic model using the stepwise method. [file TMI-25-839-s001.docx]

Supplementary material 1 - Bayesian Information Criterion results and significance values for variable selection for multivariate logistic model using the stepwise method

| Variables | BIC | p value* |
| --- | --- | --- |
| Age | 8959.5 | 0.081 * |
| Schooling | 8965.8 | 0.075 * |
| AIDS | 8970.4 | 0.985 |
| Proportion of treatment dropouts (ecological variable) | 8970.4 | 0.974 |
| Human development Index | 8970.4 | 0.972 |
| Family Health Strategy Coverage | 8970.4 | 0.966 |
| Proportion of cases diagnosed by active search | 8970.4 | 0.917 |
| Proportion of cultures grown | 8970.4 | 0.877 |
| Proportion of people with access to water supply | 8970.4 | 0.838 |
| Average Gross Domestic Product in the period | 8970.4 | 0.818 |
| Proportion of cases diagnosed in Urgency, Emergency and / or Hospitalization | 8970.5 | 0.693 |
| Degree of urbanization | 8970.6 | 0.651 |
| Proportion of population with per capita monthly nominal income up to ¼ minimum wage | 8970.7 | 0.575 |
| Municipality with prison unit | 8970.7 | 0.565 |
| Doctors per 1000 population | 8970.7 | 0.561 |
| Coverage of the Bolsa Familia Program | 8970.8 | 0.533 |
| Proportion of sputum smears performed | 8970.9 | 0.473 |
| Sex | 8970.9 | 0.468 |
| Proportion of people with access to garbage collection service | 8971.1 | 0.399 |
| Proportion of Directly Observed Treatments Performed | 8971.2 | 0.362 |
| Demographic density | 8971.2 | 0.356 |
| Proportion of Sensitivity Tests Performed | 8971.4 | 0.299 |
| Total inhabitants | 8971.7 | 0.255 |
| Public expenditure on health per inhabitant | 8971.8 | 0.237 |
| Mental disease | 8971.8 | 0.225 |
| Proportion of people with access to sewage service | 8972.1 | 0.193 |
| Smoking | 8972.2 | 0.170 |
| Primary Care Coverage | 8972.8 | 0.119 |
| Nurses per thousand inhabitants | 8973 | 0.102 |
| Alcoholism | 8973,6 | 0.074 * |
| Illicit drug use | 8974.6 | 0.039 * |
| Nursing assistants per thousand inhabitants | 8974.6 | 0.038 * |
| Nursing technicians per thousand inhabitants | 8974.8 | 0.036 * |
| Clinical form of tuberculosis | 8975.2 | 0.027 * |
| Diagnostic Form | 8978.7 | 0.003 * |
| Ethnicity | 8979.2 | > 0.001 * |
| Proportion of population with per capita monthly nominal income of ¼ to ½ minimum wage | 8981.7 | > 0.001 * |
| Proportion of cases diagnosed after death | 8983,5 | > 0.001 * |
| X-ray exam result | 8986 | > 0.001 * |
| Gini Index | 8987.9 | > 0.001 * |
| Family Health Strategy Coverage | 8992.2 | > 0.001 * |
| Proportion of HIV tests performed | 8992.6 | > 0.001 * |
| Place of residence | 9006.4 | > 0.001 * |
| Diabetes mellitus | 9014.5 | > 0.001 * |
| Date of diagnosis | 9072.6 | > 0.001 * |
| Culture examination result | 9389.4 | > 0.001 * |
| Previous History of Tuberculosis Treatments | 10049.1 | > 0.001 * |

* Selection criteria: p values >0.1
